# Optimizing Surgical Outcomes: The Role of Enhanced Recovery After Surgery (ERAS) Protocols in Improving Recovery and Reducing Hospital Stays in Pakistan

**DOI:** 10.7759/cureus.76713

**Published:** 2025-01-01

**Authors:** Talha Amjad, Muhammad Ali Malana, Muhammad Shah Nawaz Khan, Syed Asad Hasan, Shah Fahad, Maithem Haider

**Affiliations:** 1 Anatomy, Hazrat Bari Imam Sarkar (HBS) Medical and Dental College, Islamabad, PAK; 2 General Surgery, Pakistan Institute of Medical Sciences (PIMS), Islamabad, PAK; 3 Physiology, Hazrat Bari Imam Sarkar (HBS) Medical and Dental College, Islamabad, PAK; 4 Biochemistry, Hazrat Bari Imam Sarkar (HBS) Medical and Dental College, Islamabad, PAK

**Keywords:** eras protocols, hospital stays, pakistan, postoperative complications, surgical outcomes

## Abstract

Background

Enhanced recovery after surgery (ERAS) procedures are increasingly recognized for their ability to improve surgical outcomes and shorten hospital stays, particularly in resource-constrained environments like Pakistan. This study underscores the transformative potential of ERAS protocols in not only enhancing patient recovery but also optimizing healthcare resource utilization in such settings. The research aimed to evaluate the effectiveness of ERAS protocols in reducing postoperative complications and hospital stays among surgical patients in Pakistan, with a focus on their impact on overall recovery and healthcare efficiency.

Methodology

A prospective cohort design was employed, recruiting 400 adult patients aged 18 years and older undergoing elective surgical procedures from January to December 2023. Data on demographics, adherence to ERAS procedures, postoperative complications, duration of hospital stay, and recovery metrics were gathered before, during, and after surgery for participants who were split into ERAS and standard care groups. To compare results between groups, statistical procedures such as t-tests and chi-square tests were used.

Results

The ERAS group exhibited a significantly shorter mean length of hospital stay (3.52 ± 1.27 days) compared to the standard care group (6.29 ± 2.15 days, p < 0.001). Full recovery rates at 30 days, three months, and six months were significantly higher in the ERAS group, with 160 patients (80.0%), 178 patients (89.0%), and 191 patients (95.5%), respectively. In contrast, the standard care group showed recovery rates of 122 patients (61.0%), 147 patients (73.5%), and 164 patients (82.0%) at the same intervals. The ERAS group also had lower rates of postoperative complications, with 176 patients (88.0%) experiencing no complications compared to 152 patients (76.0%) in the standard care group, and lower readmission rates, with nine patients (4.5%) compared to 19 patients (9.5%) in the standard care group.

Conclusion

The successful implementation of ERAS procedures in Pakistan highlights their potential to improve healthcare efficiency by increasing surgical recovery and lowering hospital stays.

## Introduction

Enhanced recovery after surgery (ERAS) procedures have been widely acknowledged as a revolutionary approach to surgical treatment in recent years [1]. The goal of these evidence-based perioperative care pathways is to enhance patient outcomes by lowering the psychological and physical stress that comes with surgery [2]. ERAS procedures seek to minimize problems, decrease recovery periods, and shorten hospital stays by improving the preoperative, intraoperative, and postoperative stages of surgical therapy [3]. ERAS protocols were first created for colorectal surgery, but they have now been modified for use in other surgical specialties, indicating that they have the potential to improve recovery for a wide range of operations [4].

It is impossible to overestimate the significance of ERAS procedures in healthcare systems that are beset by a lack of resources and overworked hospitals, such as those in Pakistan [5]. A high patient-to-healthcare provider ratio, congested hospitals, and a lack of access to cutting-edge medical technology are just a few of the major issues facing Pakistan's healthcare system [6]. Due to problems, poor postoperative care, and delayed recovery, surgical patients often have to remain in the hospital for extended periods of time in such a setting, which puts an additional burden on the healthcare system [7].

Pakistan's use of ERAS procedures has great potential to enhance surgical results and reduce the strain on medical facilities [8,9]. In order to promote a quicker recovery, the guidelines place a strong emphasis on patient-centered care, which includes early movement, optimal nutrition, and efficient pain management [10]. In a healthcare system with limited resources, using these routes may result in a considerable reduction in hospital stay length and a decrease in postoperative complications [11].

The adoption of ERAS protocols in Pakistan's healthcare system is still in its early stages, and there is little information available about their efficacy in the local setting, despite the potential advantages. Additionally, there is still a great deal of study to be done on how to properly modify ERAS procedures to meet the particular difficulties faced by Pakistan's healthcare system. Context-specific research is required to assess the feasibility of ERAS in this setting due to a lack of resources, infrastructure limitations, and varied standards of care.

Research objective

This research aimed to investigate the effectiveness of ERAS protocols in reducing postoperative complications and hospital stays among surgical patients in Pakistan, evaluating their impact on overall recovery and healthcare efficiency.

## Materials and methods

Study design and setting

This study employed a prospective cohort design conducted at the Hazrat Bari Imam Sarkar (HBS) Medical and Dental College, Islamabad, Pakistan, from January 2023 to December 2023, with the aim of evaluating the effectiveness of ERAS protocols in improving postoperative outcomes and optimizing healthcare efficiency. The intervention involved the use of an ERAS or modified ERAS protocol, which includes preoperative, intraoperative, and postoperative interventions in line with the guidelines set forth by the ERAS Society [12]. These protocols are designed to enhance recovery and minimize complications through strategies such as multimodal anesthesia, early mobilization, optimal nutrition, and minimal use of drains and catheters. Studies reporting such protocols were considered adequate for inclusion in this study. Given the complex nature of ERAS interventions, each protocol from the included studies was narratively described.

Inclusion and exclusion criteria

Adult patients who were 18 years of age or older, had elective surgery, gave their informed permission to participate, and followed ERAS guidelines were included in this research. People without serious comorbidities that may hinder their recuperation, including severe respiratory or cardiovascular conditions, were also included. On the other hand, patients having a history of severe anesthetic or postoperative drug allergic responses, those in urgent need of surgery, and those incapable of giving informed permission or following ERAS procedures were not included in the research.

Sample size

The sample size was determined using the World Health Organization's methodology for sample size calculation in health research. A total of 400 participants were recruited to ensure adequate statistical power for detecting significant differences in outcomes. This calculation was based on a 95% confidence level, a 5% margin of error, and an anticipated effect size derived from preliminary data.

Data collection

Standardized forms (Appendix 1) were used for preoperative, intraoperative, and postoperative data collection. Demographics, surgical techniques, ERAS protocol compliance, postoperative complications, duration of hospitalization, and recovery metrics were among the data. To monitor adherence to ERAS protocols, compliance was assessed using detailed checklists for each protocol phase, which were completed by healthcare professionals involved in patient care. At 30 days, three months, and six months after surgery, patients' recovery results and complications were tracked.

Monitoring adherence to ERAS protocols

To ensure adherence to ERAS protocols, a comprehensive monitoring system was implemented. Detailed checklists were developed for each phase of the ERAS protocol: preoperative, intraoperative, and postoperative. These checklists outlined specific interventions and milestones, such as preoperative counseling, carbohydrate loading, multimodal analgesia, early mobilization, and nutritional support. Healthcare professionals directly involved in patient care completed these checklists during routine clinical evaluations. Adherence data were reviewed regularly to identify deviations or challenges in protocol implementation. In addition, multidisciplinary team meetings were held weekly to discuss adherence levels and address any barriers to compliance.

Statistical analysis

IBM SPSS Statistics for Windows, Version 27 (Released 2020; IBM Corp., Armonk, New York, United States) was used to examine the data. Clinical and demographic features were summed together using descriptive statistics. Chi-square tests for categorical variables and t-tests for continuous variables, if applicable, were used to compare the results of ERAS protocol participants with those of conventional treatment. P-values below 0.05 were regarded as statistically significant.

Ethical approval

The Institutional Review Board (IRB) of HBS Medical and Dental College granted ethical clearance for the research before any participants were recruited. All participants gave their informed permission, guaranteeing that they were aware of the goals, methods, possible dangers, and advantages of the research.

## Results

The average age of patients in both groups was similar, with the standard care group having an average age of 46.84 ± 11.91 years and the ERAS group at 45.32 ± 12.47 years, as shown in Table 1. The gender distribution was nearly the same, with 103 (51.5%) men in the ERAS group and 105 (52.5%) men in the standard care group. The average body mass index (BMI) was slightly lower in the ERAS group at 27.60 ± 4.50 kg/m² compared to 28.10 ± 4.20 kg/m² in the standard care group. Surgical procedures included colorectal (51 ERAS, 47 standard care), abdominal (69 ERAS, 66 standard care), orthopedic (37 ERAS, 41 standard care), gynecological (23 ERAS, 27 standard care), and urological (20 ERAS, 19 standard care). Postoperative pain scores were significantly lower in the ERAS group (3.23 ± 1.58) compared to the standard care group (5.81 ± 1.89). Additionally, 88% of patients in the ERAS group had no complications, compared to 76% in the standard care group. Readmission rates within 30 days were lower in the ERAS group (4.5%) compared to the standard care group (9.5%), and the average length of hospital stay was shorter in the ERAS group (3.52 ± 1.27 days) compared to the standard care group (6.29 ± 2.15 days). The ERAS protocol significantly improved postoperative outcomes, including reduced pain, complications, readmissions, and hospital stays.

**Table 1:** Patient characteristics, surgical details, ERAS compliance, and postoperative recovery metrics ERAS: enhanced recovery after surgery

Variable		ERAS Group (n = 200)	Standard Care Group (n = 200)
Age (years)	Mean ± SD	45.32 ± 12.47	46.84 ± 11.91
Gender	Male	103 (51.50%)	105 (52.50%)
	Female	97 (48.50%)	95 (47.50%)
Body Mass Index (kg/m²)	(Mean ± SD)	27.60 ± 4.50	28.10 ± 4.20
Surgical Procedure	Colorectal Surgery	51 (25.50%)	47 (23.50%)
	Abdominal Surgery	69 (34.50%)	66 (33.00%)
	Orthopedic Surgery	37 (18.50%)	41 (20.50%)
	Gynecological Surgery	23 (11.50%)	27 (13.50%)
	Urological Surgery	20 (10.00%)	19 (9.50%)
Adherence to ERAS Protocol	Full Adherence	173 (86.50%)	N/A
	Partial Adherence	27 (13.50%)	N/A
Postoperative Pain Score (0-10)	(Mean ± SD)	3.23 ± 1.58	5.81 ± 1.89
Postoperative Complications	No Complications	176 (88.00%)	152 (76.00%)
	Minor Complications	15 (7.50%)	29 (14.50%)
	Major Complications	9 (4.50%)	19 (9.50%)
Length of Hospital Stay (days)	(Mean ± SD)	3.52 ± 1.27	6.29 ± 2.15
Recovery Metrics	Early Mobilization (within 24 hours)	180 (90.00%)	120 (60.00%)
	Discharge within 5 days	160 (80.00%)	122 (61.00%)
	Readmission within 30 days	9 (4.50%)	19 (9.50%)

The ERAS group demonstrated superior outcomes compared to the standard care group across 30 days, three months, and six months post-surgery, as shown in Table 2. At 30 days, 160 (80.0%) ERAS patients were fully recovered, compared to 122 (61.0%) in the standard care group. Full recovery rates increased further in the ERAS group, reaching 178 (89.0%) at three months and 191 (95.5%) at six months, while the standard care group showed recovery rates of 147 (73.5%) at three months and 164 (82.0%) at six months. Minor complication rates in the ERAS group were 15 (7.5%) at 30 days, decreasing to two (1.0%) by six months. In comparison, the standard care group had 29 (14.5%) minor complications at 30 days, which reduced to 12 (6.0%) by six months. Major complications at 30 days were reported in nine (4.5%) ERAS patients, compared to 19 (9.5%) in the standard care group. Furthermore, readmission rates within 30 days were lower in the ERAS group (4.5%) compared to the standard care group (9.5%), and reoperation rates were also lower in the ERAS group at 2.0%, compared to 3.0% in the standard care group. These results highlight the effectiveness of the ERAS protocol in improving recovery, reducing complications, and minimizing readmission and reoperation rates.

**Table 2:** Post-surgery recovery outcomes and complications at 30 days, three months, and six months for ERAS and standard care groups ERAS: enhanced recovery after surgery

Outcome/Complication	Time Point	ERAS Group (n = 200)	Standard Care Group (n = 200)
Full Recovery	30 Days	160 (80.00%)	122 (61.00%)
	3 Months	178 (89.00%)	147 (73.50%)
	6 Months	191 (95.50%)	164 (82.00%)
Minor Complications	30 Days	15 (7.50%)	29 (14.50%)
	3 Months	10 (5.00%)	18 (9.00%)
	6 Months	2 (1.00%)	12 (6.00%)
Major Complications	30 Days	9 (4.50%)	19 (9.50%)
	3 Months	5 (2.50%)	13 (6.50%)
	6 Months	1 (0.50%)	8 (4.00%)
Readmission	30 Days	9 (4.50%)	19 (9.50%)
	3 Months	5 (2.50%)	13 (6.50%)
	6 Months	1 (0.50%)	8 (4.00%)
Reoperation Required	30 Days	4 (2.00%)	6 (3.00%)
	3 Months	4 (2.00%)	5 (2.50%)
	6 Months	1 (0.50%)	8 (4.00%)
Mortality	30 Days	0 (0.00%)	1 (0.50%)
	3 Months	0 (0.00%)	1 (0.50%)
	6 Months	0 (0.00%)	2 (1.00%)

Statistical analysis confirmed significant differences between the groups, as shown in Table 3. The mean duration of hospital stay was significantly shorter in the ERAS group at 3.52 ± 1.27 days, compared to 6.29 ± 2.15 days in the standard care group, with a t-test statistic of -9.50 (p < 0.001). Full recovery rates at six months were notably higher in the ERAS group, with 191 (95.5%) fully recovered, compared to 164 (82.0%) in the standard care group (chi-square = 19.50, p < 0.001). Minor complications were also significantly lower in the ERAS group (15 (7.5%)) versus standard care (29 (14.5%)), with a chi-square value of 6.65 (p = 0.010). Although major complications were fewer in the ERAS group (9 (4.5%)) compared to the standard care group (19 (9.5%)), the difference did not reach statistical significance (chi-square = 3.54, p = 0.060). These statistical findings further support the effectiveness of the ERAS protocol in improving recovery and reducing complications.

**Table 3:** Comparative outcomes of ERAS and standard care: t-test and chi-square analyses ERAS: enhanced recovery after surgery

Outcome Variable	ERAS Group (n = 200)	Standard Care Group (n = 200)	Statistical Test	Statistic Value	p-value
Length of Hospital Stay (days) (Mean ± SD)	3.52 ± 1.27	6.29 ± 2.15	t-test	-9.50	<0.001
Full Recovery (%)	191 (95.50%)	164 (82.00%)	Chi-square	19.50	<0.001
Minor Complications	15 (7.50%)	29 (14.50%)	Chi-square	6.65	0.010
Major Complications	9 (4.50%)	19 (9.50%)	Chi-square	3.54	0.060

## Discussion

The use of ERAS protocols has shown encouraging benefits in terms of improving surgical outcomes, especially in countries with low resources such as Pakistan. With a p-value of less than 0.001, we discovered that the ERAS group's mean duration of hospital stay was substantially lower (3.52 ± 1.27 days) than that of the standard care group (6.29 ± 2.15 days). These findings are consistent with other research that found that patients who adhered to ERAS standards had shorter hospital stays, suggesting that ERAS deployment may result in more effective healthcare delivery and lower resource use in surgical wards [13,14].

Furthermore, our results show that the ERAS group had a greater rate of full recovery at 30 days after surgery (80.0%) than the standard care group (61.0%). This trend persisted at three and six months (89.0% vs. 73.5% and 95.5% vs. 82.0%), demonstrating the efficiency of ERAS in hastening patient recovery. This supports our findings and implies that ERAS may be a flexible strategy in a variety of surgical scenarios [15]. Other research has also shown that ERAS protocols considerably improve recovery rates after a range of surgical procedures.

Along with better recovery rates, our research showed that the ERAS group saw significantly fewer postoperative problems, just 7.5% experienced mild difficulties, compared to 14.5% in the standard care group (p = 0.010). Additionally, the ERAS group had considerably fewer serious problems (4.5% vs. 9.5%, p = 0.060). These results are consistent with other research that has shown that ERAS procedures reduce postoperative complications by emphasizing early mobility and multimodal analgesia, two crucial ERAS framework elements [16,17]. According to their research, lowering complications lowers total healthcare expenses while simultaneously increasing patient satisfaction. This is especially important in Pakistan's healthcare system, which has limited resources.

Furthermore, compared to 9.5% of patients in the standard care group, only 4.5% of ERAS patients were readmitted within 30 days, according to our data. The efficiency of ERAS in enhancing postoperative care and follow-up is shown by this lower readmission rate, which is corroborated by the results of the prior meta-analysis [18]. According to the meta-analysis, following ERAS guidelines might reduce the chance of readmissions by making sure patients are ready for release and have the support networks they need to recover.

Surgical site infections (SSIs) are a major concern following colorectal surgery, as they are associated with increased pain, prolonged hospital stays, readmission rates, and economic burdens. Our study acknowledges the role of ERAS in potentially reducing SSIs by optimizing perioperative care. ERAS protocols improve wound healing and minimize inflammation, which are critical factors in reducing the risk of SSIs. Studies have consistently shown that adherence to ERAS protocols leads to lower rates of SSIs, partly due to enhanced patient mobility, optimal nutritional support, and reduced postoperative opioid use [19,20]. Additionally, recent studies have identified butyrylcholinesterase (BChE) levels as a potential predictive marker for postoperative complications, including SSIs. Low levels of BChE have been associated with higher risks of developing SSIs and anastomotic leaks. Integrating BChE monitoring into the ERAS framework could refine patient management by identifying those at higher risk of infection and allowing for early intervention [21].

Study strengths and limitations

The prospective cohort form of this research improves the trustworthiness of the results, and the large sample size of 400 patients enables statistical comparisons between the ERAS and standard care groups. Furthermore, a thorough grasp of recovery trajectories is provided by the extensive data collection at various postoperative time points. The study's single-center design is one of its drawbacks, however, since it would restrict how broadly the findings can be applied to other Pakistani healthcare environments. Additionally, results may be impacted by possible biases in patient selection and ERAS procedure adherence; therefore, care must be used when extrapolating results to larger populations.

## Conclusions

There is considerable promise for improving surgical outcomes in Pakistan with the use of ERAS protocols, particularly in the context of limited healthcare resources. Our research demonstrated that ERAS procedures result in significantly shorter hospital stays, with the ERAS group having an average stay of 3.52 ± 1.27 days compared to 6.29 ± 2.15 days in the standard care group. Additionally, ERAS patients experienced better recovery rates, with 80.0% of patients fully recovered at 30 days post-surgery, compared to 61.0% in the standard care group. This trend continued at three and six months, showing recovery rates of 89.0% and 95.5% in the ERAS group, respectively, compared to 73.5% and 82.0% in the standard care group. Furthermore, the ERAS group reported fewer postoperative complications, with only 7.5% of patients experiencing mild issues compared to 14.5% in the standard care group, and a lower rate of major complications (4.5% vs. 9.5%). These findings highlight the potential of ERAS protocols to improve patient recovery, reduce complications, and lessen the strain on healthcare resources, making it an important strategy for Pakistan’s healthcare system, where resources are often limited.

The positive outcomes of ERAS suggest that its implementation can help alleviate pressure on Pakistan’s healthcare infrastructure by reducing hospital stays and postoperative complications, leading to more efficient care delivery and cost savings. Given the resource constraints in the country, ERAS could be a transformative solution for improving surgical care while optimizing the use of healthcare resources. However, further research is essential to explore the wider application of ERAS across different surgical specialties in Pakistan, such as cardiothoracic, urological, and neurosurgical procedures. Multi-center randomized controlled trials (RCTs) are needed to validate the broader applicability of ERAS protocols and assess how they can be tailored to fit the diverse healthcare settings across the country, ensuring that ERAS can be effectively integrated into Pakistan’s unique healthcare system.

## Appendices

Appendix 1 

**Table 4:** ERAS protocol adherence questionnaire form ERAS: enhanced recovery after surgery; NSAIDs: non-steroidal anti-inflammatory drugs; LMWH: low molecular weight heparin; IV: intravenous

Statement of Consent By participating in this study, I confirm that I have been informed about the purpose, procedures, and potential benefits of this research. I voluntarily agree to provide accurate and honest responses to the questionnaire. I understand that my responses will remain confidential and will be used solely for research purposes.				
Participant's Name (Optional): ________________________				
Signature: ________________________________				
Date: __________________________				
Section	Question	Yes	No	Comments
Preoperative Phase	Was the patient informed about the ERAS protocol?	☐	☐	
	Was psychological counseling provided (if needed)?	☐	☐	
	Was the patient screened for nutritional deficiencies (e.g., hypoalbuminemia)?	☐	☐	
	Was preoperative fasting minimized (clear liquids allowed up to 2 hours before surgery)?	☐	☐	
	Was carbohydrate loading administered 2–3 hours preoperatively?	☐	☐	
	Were prophylactic antibiotics administered within 60 minutes before incision?	☐	☐	Specify type & dose
	Was venous thromboembolism prophylaxis provided (e.g., compression stockings, LMWH)?	☐	☐	Specify method
	Were preoperative bowel preparation guidelines followed?	☐	☐	
Intraoperative Phase	Was a minimally invasive surgical approach used (if applicable)?	☐	☐	
	Was multimodal anesthesia administered (e.g., regional + general)?	☐	☐	Specify medications
	Were short-acting anesthetics used?	☐	☐	Specify agents
	Was goal-directed fluid therapy implemented (e.g., intraoperative fluid balance maintained)?	☐	☐	Details on fluids
	Was the patient’s body temperature maintained throughout the procedure (e.g., active warming)?	☐	☐	Specify method
	Were prophylactic antiemetics provided to prevent postoperative nausea/vomiting?	☐	☐	
	Was the surgical site protected using sterile techniques (e.g., wound protectors)?	☐	☐	
Postoperative Phase	Was the patient mobilized on the day of surgery or postoperative day 1?	☐	☐	Details on timing
	Was a high-protein diet or nutritional supplementation provided early postoperatively?	☐	☐	
	Were fluids introduced early (oral or IV)?	☐	☐	Specify type
	Was opioid-sparing analgesia used (e.g., epidural, nerve block)?	☐	☐	Specify method
	Were non-opioid analgesics (e.g., NSAIDs or acetaminophen) provided?	☐	☐	Specify medications
	Were drains, catheters, or nasogastric tubes minimized or removed early?	☐	☐	Specify timing
	Was the patient given respiratory physiotherapy (e.g., incentive spirometry)?	☐	☐	
	Were wound care and infection prevention measures followed (e.g., daily dressings)?	☐	☐	Specify approach
	Was patient education on postoperative recovery provided (e.g., warning signs, discharge plan)?	☐	☐	
Follow-Up	Was the patient assessed for complications within 30 days of surgery?	☐	☐	Specify findings
	Was a follow-up appointment scheduled within 3–6 months post-surgery?	☐	☐	
